# AquaAI: development and internal validation of a Danish transformer-based model to identify drowning and aquatic incidents in prehospital medical records

**DOI:** 10.1186/s13049-026-01644-y

**Published:** 2026-06-09

**Authors:** Caroline Kamuk Ostenfeldt, Niklas Breindahl, Claus Graff, Kristian Hay Kragholm, Helle Collatz Christensen, Stig Nikolaj Fasmer Blomberg, the Danish Drowning Validation Group

**Affiliations:** 1 Prehospital Center, Region Zealand, Naestved, Denmark; 2 Department of Health Science and Technology, Aalborg University, Aalborg, Denmark; 3 Department of Applied Mathematics and Computer Science, Technical University of Denmark, Copenhagen, Denmark; 4 Department of Clinical Medicine, University of Copenhagen, Copenhagen, Denmark

**Keywords:** Drowning, Near-drowning, Prehospital, Electronic medical record, Natural language processing, Artificial intelligence, Machine learning, BERT, Surveillance, Denmark

## Abstract

**Background:**

Effective prevention of drowning and aquatic incidents requires timely and accurate surveillance supported by high-quality validated data. In Denmark, the use of the free-text fields in the Danish Prehospital Medical Record has proven effective in identifying potentially relevant cases for such surveillance. While these free-text fields contain rich contextual information, manual screening of all records is impractical. This study aimed to develop and internally validate a Danish natural language processing pipeline for identifying drowning and aquatic incidents (AquaAI) from routine prehospital records and prioritizing records for final manual validation by medical experts.

**Methods:**

This nationwide retrospective cohort study was conducted using Danish prehospital electronic medical records from 2016 to 2024. Medical records were first retrieved using the Danish Drowning Formula, an iteratively developed trigger-word search algorithm, and expert-labelled as Drowning, Aquatic incident, or Non-relevant. A Danish transformer-based language model was then fine-tuned for three-class sequence classification as the second-stage classifier in this workflow and embedded in a hybrid pipeline with rule-based safeguards. Model development used a temporal split with training data from 2016 to 2021 and validation data from 2022. Final performance was evaluated on a temporally separated hold-out dataset comprising records from 2023 to 2024. Primary outcomes were class-wise sensitivity and a binary Relevant (Drowning + Aquatic incident) versus Non-relevant analysis to quantify case finding and workload reduction.

**Results:**

The dataset comprised 40,876 medical records retrieved with the Danish Drowning Formula. On the hold-out dataset, AquaAI identified 91% of relevant cases and correctly filtered 89% of non-relevant records from manual review. Overall, this reduced the number of records requiring manual review by 77.5%. In the three-class analysis, sensitivity was 84% for Drowning, 83% for Aquatic incident, and 89% for Non-relevant. Only one drowning case (0.3%) was classified as non-relevant.

**Conclusions:**

A hybrid transformer-based pipeline operating as a second-stage classifier within a two-step retrieval workflow can identify drowning and aquatic incidents from prehospital free-text narratives with high case finding and substantial reduction of manual review. This approach may support national surveillance and targeted prevention initiatives.

**Supplementary Information:**

The online version contains supplementary material available at 10.1186/s13049-026-01644-y.

## Background

Denmark’s extensive coastline, harbors, and public bathing facilities create frequent population exposure to aquatic environments and associated risk of drowning and other water-related incidents. Drowning is a preventable cause of death [1], and effective prevention depends on timely, accurate, and comprehensive surveillance data [2].

In this study, Drowning refers to incidents involving respiratory impairment from submersion or immersion in liquid, whereas Aquatic incident denotes water-related incidents without evidence of respiratory impairment, a distinction aligned with internationally accepted definitions [3, 4].

Recent Danish drowning statistics relied mainly on International Classification of Diseases, 10th Revision (ICD-10) coding from death certificates [5]. Although useful for mortality surveillance, this approach is limited because it is likely to underestimate the full burden of drowning, particularly non-fatal events, and provides limited detail on the broader spectrum of aquatic incidents relevant to prevention [6–8].

Since 2015, the Danish emergency medical services across all regions have documented care in a nationwide electronic prehospital medical record which includes multiple free-text narrative fields describing scene circumstances, patient condition, and treatment [9–11]. Structured variables in the prehospital record, such as age, sex, vital signs, and predefined response categories, do not capture the circumstantial and contextual information required to identify drowning and aquatic incidents. Instead, the circumstances of water exposure, presence or absence of respiratory impairment, bystander observations, and scene descriptions are predominantly documented in free-text narrative fields, making these fields indispensable for surveillance purposes.

Prior Danish work using the Danish Drowning Formula, an iteratively developed trigger-word search algorithm for identifying potentially drowning-related cases in prehospital free-text fields, has shown that such retrieval can identify potentially relevant drowning incidents and support text-based surveillance [12, 13]. However, this approach requires manual validation, which is not feasible at scale and causes significant delay in data analysis.

Advances in natural language processing, particularly transformer-based language models such as Bidirectional Encoder Representations from Transformers (BERT) [14], allow context-sensitive classification directly from raw clinical text and have shown promise in healthcare text analysis [15]. For Danish-language narratives, monolingual or Scandinavian models may offer advantages over broadly multilingual models [16, 17]. Together, these developments create an opportunity to move from keyword-based retrieval toward automated classification of drowning and aquatic incidents in Danish routine prehospital documentation.

The aim of this study was to develop and internally validate AquaAI, a Danish natural language processing pipeline for identifying drowning and aquatic incidents from the free-text fields in the nationwide prehospital electronic medical record system, using a temporally separated hold-out dataset.

## Methods

### Study design

This was a nationwide retrospective registry-based cohort study. This study is reported in accordance with the Strengthening the Reporting of Observational Studies in Epidemiology (STROBE) statement for observational studies [18] and, given the AI classification component, additionally in accordance with the Transparent Reporting of a multivariable prediction model for Individual Prognosis Or Diagnosis with Artificial Intelligence (TRIPOD + AI) reporting guideline for studies developing and validating AI-based prediction models [19].

### Setting

Denmark has approximately 6 million inhabitants and is surrounded by extensive coastal and inland aquatic environments, creating broad population exposure to water-related incidents [20, 21].

Prehospital Emergency Medical Services are publicly funded and organized across five administrative regions, each with its own emergency medical dispatch center operating 24/7 [9, 10]. Since 2015, all five regions have used a nationwide electronic prehospital electronic medical record system that includes both structured variables and several Danish free-text fields documenting scene circumstances, patient condition, and treatment interventions. These free-text narratives are entered during routine care and forwarded to receiving hospitals [9].

### Data source and cohort assembly

A nationwide dataset of prehospital electronic medical record narratives for incidents dated 1 January 2016 to 31 December 2024 was assembled for analysis. Candidate records were retrieved using the Danish Drowning Formula, an iteratively developed trigger-word search algorithm previously shown to identify potentially drowning-related cases in prehospital free-text fields [13]. The retrieved records were manually annotated by trained investigators to create the labelled dataset used for model development and evaluation. Detailed data collection and annotation procedures have been reported elsewhere [12].

### Target outcome definition

Each prehospital record identified in the nationwide electronic prehospital medical record system was assigned one of three mutually exclusive labels aligned with surveillance needs: Drowning, Aquatic incident, or Non-relevant. Annotation was conducted in accordance with the internationally accepted definition of drowning as “the process of experiencing respiratory impairment from submersion or immersion in liquid” [3], supported by the clarification statement for non-fatal drowning [4]. Drowning outcomes are classified as fatal or non-fatal, with non-fatal drowning defined as interruption of the respiratory impairment process before death occurs [4]. Both fatal and non-fatal drowning incidents were included, including patients declared dead in the prehospital setting and patients with obvious clinical signs of irreversible death. Aquatic incident denoted incidents involving submersion or immersion in liquid without evidence of respiratory impairment. Non-relevant comprised records unrelated to drowning or aquatic exposure that had been retrieved by the Danish Drowning Formula because of its broad search criteria [12, 13].

### Validation strategy

A strict temporal split by incident year was applied to the dataset. Records from calendar years 2016 to 2021 formed the training set (60%), and calendar year 2022 was reserved a priori as the validation set (13%) for model selection and specification of the final operating configuration. Final model performance was evaluated on a temporally separated hold-out dataset comprising records from calendar years 2023 and 2024 (26%). This split was chosen to preserve a large development sample while reserving a substantial, fully unseen and temporally later dataset for final evaluation. All splits were assigned by incident date before modelling and remained fixed throughout. The final model, thresholds, and rule-based safeguards were applied to the hold-out dataset without any additional training or tuning.

### BERT fine-tuning and model development

Maltehb/danish-bert-botxo [22], a Danish transformer language model based on the BERT architecture [14] and pre-trained on large-scale Danish text, was used as the backbone for sequence classification. The pre-trained model was fine-tuned on the annotated training narratives for three-class sequence classification using the Hugging Face Trainer API with AdamW optimization (learning rate 2 × 10⁻⁵, batch size 16, maximum 10 epochs with early stopping based on validation F1-score). Class-weighted cross-entropy loss was used to address class imbalance. This choice of model was supported by prior benchmarking of Scandinavian and Danish language models in downstream NLP tasks [16, 23]. Full technical details are specified in supplementary materials [see Additional file 1].

Before fine-tuning, the free-text fields were concatenated in a fixed order to preserve context. Long texts were truncated to the model input limit before tokenization. No imputation was performed, as the analysis was based on available narrative text only. All included records contained at least one non-empty narrative field. Only minimal preprocessing was applied and no stemming or stop-word removal was used. To reduce imbalance during training, all Drowning and Aquatic incident narratives were retained, whereas the Non-relevant class was randomly undersampled in the training set only to a prespecified cap of 5,000 records using a fixed random seed. The validation set (2022) and the temporally separated hold-out dataset (2023–2024) were kept at natural prevalence without resampling or augmentation.

**Fig. 1:**
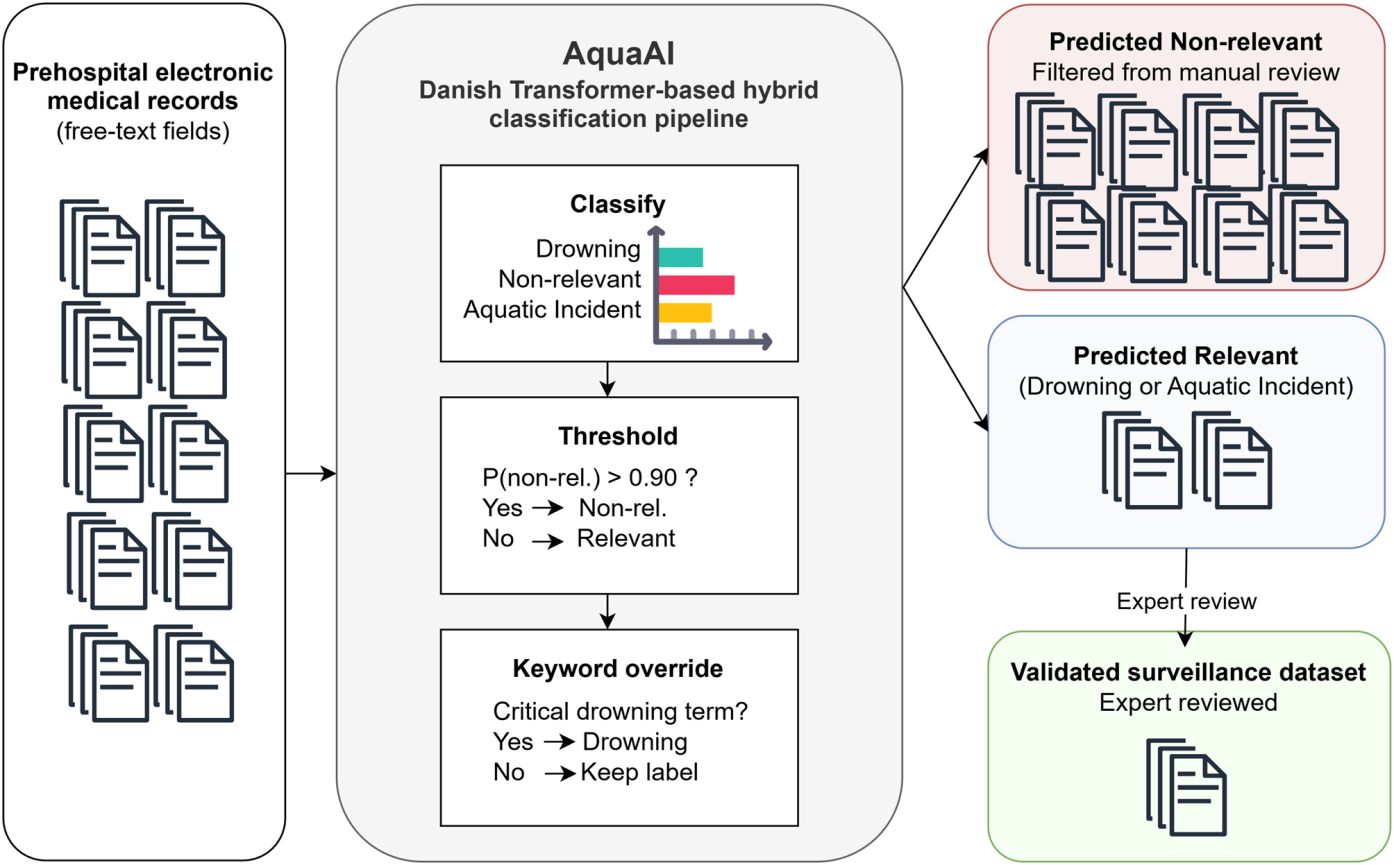
Overview of the AquaAI hybrid classification pipeline. Prehospital free-text records were processed through the AquaAI pipeline, which combined transformer-based three-class classification with a prespecified threshold for assigning non-relevant records and a keyword override for critical drowning terms. Records predicted as Non-relevant were filtered from manual review, whereas records predicted as Relevant (Drowning or Aquatic incident) were forwarded for expert review, resulting in an expert-validated surveillance dataset

The overall AquaAI classification pipeline is illustrated in Fig. 1. The classifier was embedded in a hybrid decision pipeline designed to minimize missed drownings while reducing unnecessary manual review. First, a record was assigned directly to Non-relevant only if the model probability for that class exceeded a prespecified threshold. Threshold candidates were evaluated on the 2022 validation set and the final operating threshold of 0.90 was selected based on the Youden index. Full threshold comparison including receiver operating characteristic (ROC) and precision-recall curves is provided in Additional file 4. When the Non-relevant probability did not exceed this threshold, the record was routed to the most probable relevant class (Aquatic incident or Drowning). Second, an expert-curated critical-keyword override reclassified cases initially identified as Non-relevant to Drowning when predefined drowning-related terms or phrases were detected in the text. Overrides were one-directional and logged as audit flags. Full keyword lists and matching rules are provided in supplementary materials [see Additional file 2].

### Performance measures and evaluation strategy

Primary evaluation was performed on the temporally separated hold-out dataset comprising records from calendar years 2023 and 2024. Performance was reported as a three-class confusion matrix (a table cross-tabulating predicted against reference labels across all three classes) for Drowning, Aquatic incident, and Non-relevant, with class-wise sensitivity for each category. As the intended use of the model is to prioritize records for manual expert validation, a binary 2 × 2 analysis is also reported, with Drowning and Aquatic incident combined into Relevant and compared with Non-relevant. The following metrics were derived: sensitivity (true positive rate, TP/(TP + FN)); specificity (true negative rate, TN/(TN + FP)); positive predictive value (PPV, TP/(TP + FP)); negative predictive value (NPV, TN/(TN + FN)); F1-score (harmonic mean of sensitivity and PPV); accuracy ((TP + TN)/N); and predicted negative rate, reflecting the proportion of all records removed from manual review ((TN + FN)/N). In operational terms, specificity reflects the proportion of Non-relevant records correctly filtered from manual review, and the predicted negative rate reflects the overall workload reduction achieved by AquaAI. The prespecified primary outcome was the combined sensitivity for Relevant cases. For all primary performance measures, 95% confidence intervals were calculated using the Wilson score method. As a safety-oriented secondary measure, the number of Drowning cases classified as Non-relevant is reported. The area under the receiver operating characteristic curve (AUC-ROC) and the area under the precision-recall curve (AUC-PR) were calculated for the binary Relevant/Non-relevant classification on both the validation and hold-out datasets with 95% bootstrap confidence intervals (2,000 iterations). Given the class imbalance, AUC-PR is reported alongside AUC-ROC as a complementary summary of discrimination. Validation results are provided in Additional file 4. Operating thresholds and rule-based safeguards were fixed a priori based on the 2022 validation set and were not modified during hold-out evaluation.

### Error analysis

To further characterize model errors, a subset of misclassified cases from the hold-out dataset underwent structured manual review. All Drowning cases classified as Non-relevant were included, whereas cases from the other misclassification groups were sampled for review.

## Results

### Cohort

**Fig. 2:**
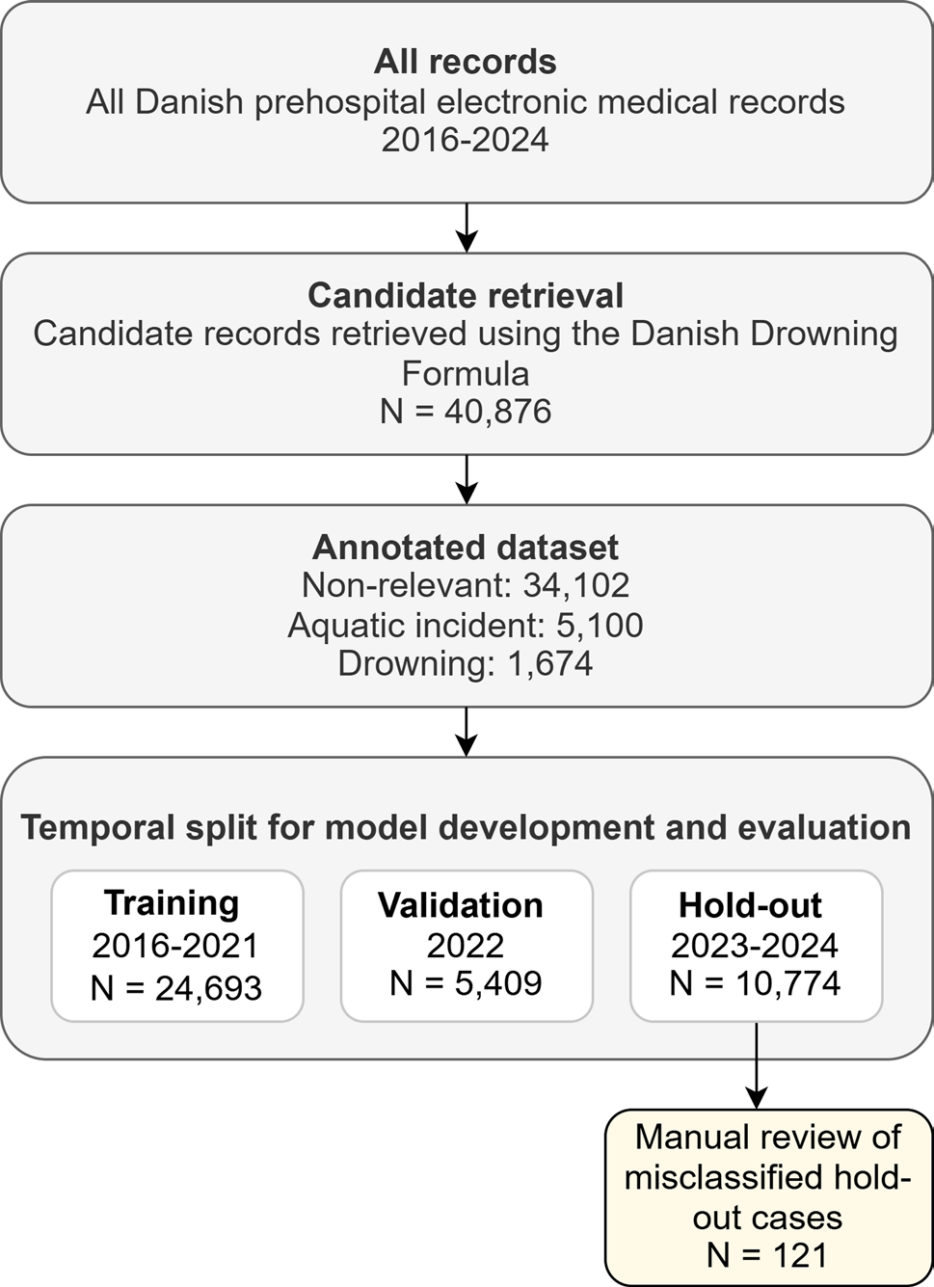
Cohort construction and temporal dataset split. Danish prehospital electronic medical record free-text narratives from 2016–2024 were first retrieved using the Danish Drowning Formula and then manually annotated as Non-relevant, Aquatic incident, or Drowning. The annotated dataset was split temporally into training (2016–2021), validation (2022), and hold-out (2023–2024) sets. A subset of misclassified hold-out cases underwent structured manual review

Application of the Danish Drowning Formula to nationwide electronic prehospital medical records from 2016 to 2024 yielded a candidate set that was manually labelled into three mutually exclusive classes. The final dataset comprised 40,876 records: 34,102 annotated as Non-relevant, 5,100 annotated as Aquatic incident, and 1,674 annotated as Drowning (Table 1). A temporal split was used for model development, with 24,693 records from 2016 to 2021 assigned to training and 5,409 records from 2022 reserved for validation. Final evaluation was performed on a temporally separated hold-out dataset comprising 10,774 records from 2023 to 2024. Dataset assembly and temporal splitting are shown in Table 1; Fig. 2.

**Table 1:** Cohort overview and class distribution by dataset split

Split (incident years)	Total *n*	Non-relevant *n* (%)	Aquatic incident *n* (%)	Drowning *n* (%)
Overall (2016–2024)	40,876	34,102 (83.5)	5100 (12.5)	1674 (4.1)
Training (2016–2021)	24,693	20,299 (82.2)	3204 (13.0)	1190 (4.8)
Validation (2022)	5409	4598 (85.0)	623 (11.5)	188 (3.5)
Hold-out (2023–2024)	10,774	9242 (85.8)	1239 (11.5)	293 (2.7)

Electronic prehospital medical record free-text fields retrieved with the Danish Drowning Formula (2016–2024) and manually annotated into three mutually exclusive classes. Values are presented as n (% within split). Splits reflect the modelling workflow: Training (2016–2021), Validation (2022), and Hold-out (2023–2024) for final evaluation

### Primary performance

On the hold-out dataset (comprising records from 2023 to 2024), AquaAI identified 91.2% (95% CI 89.7–92.5) of Relevant cases and correctly filtered 88.8% (95% CI 88.2–89.5) of Non-relevant records from manual review. Overall, this reduced the total manual review workload by 77.5% (95% CI 76.7–78.2). The AUC-ROC was 0.958 (95% CI 0.953–0.963) and the AUC-PR was 0.830 (95% CI 0.810–0.849) (Tables 2 and 3).

In the three-class analysis, sensitivity was 84.0% (95% CI 79.3–87.7) for Drowning, 83.1% (95% CI 80.9–85.0) for Aquatic incident, and 88.8% (95% CI 88.2–89.5) for Non-relevant (Table 2). Only one Drowning case (0.3%) was classified as Non-relevant. Year-specific confusion matrices and derived performance measures for 2023 and 2024 are provided in supplementary tables S3 and S4 [see Additional file 3]. The keyword override mechanism (described in Methods) was triggered in 294 hold-out cases and changed the predicted class from Non-relevant to Drowning in 57 cases. Of these 57 cases, one was annotated as Drowning and two as Aquatic incident in the reference standard.

**Table 2:** AquaAI three-class classification performance on the hold-out dataset (2023–2024)

**(A) Three-way confusion matrix**					
Total population *N* = 10,774		Reference Standard (Ground Truth)			Row total (AquaAI prediction)
		Drowning	Aquatic incident	Non- relevant	
AquaAI prediction (model output)	Drowning	246	76	182	504
	Aquatic incident	46	1029	850	1925
	Non- relevant	1	134	8210	8345
Column total (reference standard)		293	1239	9242	10,774 (total)

(B) Class-specific sensitivity					
Class			Sensitivity (95% CI)		
Drowning			84.0% (79.3–87.7%)		
Aquatic incident			83.1% (80.9–85.0%)		
Non-relevant			88.8% (88.2–89.5%)		

Panel (A) Three-class confusion matrix: columns represent the reference standard (ground truth), and rows represent the AquaAI prediction. Panel (B) reports class-specific sensitivity with 95% Wilson confidence intervals, calculated using a one-versus-rest approach. The hold-out dataset comprised records from calendar years 2023 and 2024

**Table 3:** AquaAI binary classification performance on the hold-out dataset (2023–2024)

**(A) Confusion matrix**				
Total population *N* = 10,774		Reference Standard (Ground Truth)		Row total (AquaAI prediction)
		Relevant (Positive)	Non-relevant (Negative)	
AquaAI prediction (model output)	Relevant	1397 (TP)	1032 (FP)	2429
	Non-relevant	135 (FN)	8210 (TN)	8345
Column total (reference standard)		1532	9242	10,774 (total)

(B) Performance metrics				
Performance metrics	Metric		Value (95% CI)	
*Accuracy and workload rates*	Prevalence		14.2%	
	Accuracy		89.2% (88.6–89.7%)	
	Predicted negative rate		77.5% (76.7–78.2%)	
*Discrimination metrics*	Sensitivity		91.2% (89.7–92.5%)	
	Specificity		88.8% (88.2–89.5%)	
	PPV		57.5% (55.5–59.5%)	
	NPV		98.4% (98.1–98.6%)	
	F1-score		70.5%	

Panel (A) Confusion matrix: columns represent the reference standard (ground truth), and rows represent the AquaAI prediction. Relevant collapses Drowning and Aquatic incident into a single positive class. Panel (B) Performance metrics: performance metrics are defined in the Methods section. Sensitivity reflects the proportion of Relevant cases correctly identified, while specificity reflects the proportion of Non-relevant records correctly filtered from manual review. PPV and NPV reflect the proportion of AquaAI predictions that were correct for each class. The predicted negative rate reflects the overall workload reduction achieved by AquaAI. Performance metrics are reported with 95% Wilson confidence intervals. Prevalence and F1-score are reported without confidence interval. The hold-out dataset comprised records from calendar years 2023 and 2024

### Error analysis

A structured expert review was performed for a subset of misclassified cases from the hold-out dataset (*n* = 121). These comprised 40 Aquatic incidents classified as Non-relevant, 40 Non-relevant cases classified as Relevant (Drowning or Aquatic incident), 20 Drowning cases classified as Aquatic incident, 20 Aquatic incident cases classified as Drowning, and one Drowning case classified as Non-relevant. As part of this review, it was also noted whether the original reference label remained supported by the narrative. In 88 cases it was, whereas in 32 cases the narrative did not sufficiently support the original annotation.

The most safety-critical error type was rare. Only one Drowning case was classified as Non-relevant, and this case was described indirectly through a search-and-rescue narrative rather than explicit documentation of submersion or respiratory impairment. Across the reviewed cases, the dominant error patterns were broad triggering by water-related wording, indirect or weakly documented water exposure, and boundary cases between Drowning and Aquatic incident.

Misclassifications between Drowning and Aquatic incident were typically seen in brief or ambiguous narratives in which respiratory impairment, aspiration, submersion, rescue circumstances, or altered consciousness were incompletely documented. Relevant cases classified as Non-relevant were generally mild, indirectly described, or lacked explicit respiratory features, whereas Non-relevant cases classified as Relevant often contained water-related vocabulary or scene descriptions without meeting the study definitions for Drowning or Aquatic incident. The observed error pattern was consistent with the prespecified 0.90 threshold for assigning the Non-relevant class.

## Discussion

### Summary of results

AquaAI was developed from routine prehospital narratives and internally validated on a temporally separated hold-out dataset. On the hold-out dataset, AquaAI identified 91% of relevant cases and correctly filtered 89% of non-relevant records from manual review, reducing the overall manual review workload by 77.5%. In the three-class analysis, sensitivity was 84% for Drowning, 83% for Aquatic incident, and 89% for Non-relevant. The selected decision threshold intentionally prioritized sensitivity for Relevant cases and resulted in only one Drowning case being classified as Non-relevant. Year-specific supplementary analyses showed broadly comparable performance across the 2023 and 2024 evaluation sets, supporting the temporal robustness of the combined hold-out findings.

### Relation to prior work

This study extends the Danish Drowning Formula, which showed that an iteratively developed trigger-word algorithm could retrieve potentially drowning-related cases from prehospital free-text narratives for registry use but still required manual validation at scale [12, 13]. By adding a context-aware transformer-based classifier and deterministic safeguards, AquaAI moves beyond retrieval alone toward a surveillance-oriented classification pipeline spanning the broader spectrum of Drowning and Aquatic incident.

The study findings are consistent with literature showing that natural language processing can extract clinically meaningful information from unstructured emergency care text and support identification and categorization of specific conditions or events in emergency and prehospital settings [24–26]. Prior drowning surveillance studies have primarily relied on coded administrative data or syndromic surveillance definitions rather than narrative text classification [6–8, 27]. AquaAI contributes at the intersection of these fields by applying clinical NLP to prehospital free-text narratives for drowning surveillance, thereby providing a more context-sensitive approach than keyword retrieval alone and a more use-case-specific application than general emergency text classification.

These findings also support the value of a hybrid design tailored to surveillance workflows. Rather than relying on unconstrained multiclass prediction alone, AquaAI combined transformer-based classification with a prespecified Non-relevant threshold and keyword-based safeguards. This design reflects the practical requirement of the surveillance workflow: avoiding rare but relevant drowning-related misses is more important than eliminating all false-positive records before review. This is consistent with other clinical NLP studies suggesting that hybrid approaches may be particularly useful when reliable case finding is prioritized over optimization of a single aggregate performance metric [28].

Alternative approaches, such as general-purpose large language models (LLMs) or retrieval-augmented generation (RAG) systems, could in principle be applied to this task. However, at the time of development, fine-tuned monolingual BERT-based models had demonstrated strong performance on Danish clinical text classification tasks [16, 17, 23], and their deterministic fine-tuning behavior and well-understood failure modes were considered advantageous in a safety-sensitive surveillance context. LLMs may offer broader semantic coverage but introduce stochastic output, higher computational cost, and reduced transparency, properties that are less suitable when auditability and reproducibility are priorities. RAG architectures, while potentially useful for knowledge-grounded tasks, were not considered appropriate for the pattern-classification task at hand. Compared with conventional registry-based approaches relying solely on structured ICD-coded data, the free-text classification approach captures a substantially richer set of case-defining contextual cues that structured fields cannot encode [6–8].

### Interpretation and implications for surveillance

AquaAI was designed to support surveillance rather than to function as a stand-alone classifier, and its performance should be interpreted in that context. The main operational requirement is to identify relevant cases while reducing unnecessary manual review. In the hold-out dataset, the system achieved high sensitivity for relevant-case identification while filtering a large proportion of non-relevant narratives, suggesting that transformer-based classification of prehospital narratives may support surveillance of drowning and aquatic incidents. This balance depended on the hybrid design: the transformer model contributed context sensitivity in heterogeneous narratives, whereas the prespecified Non-relevant threshold and keyword safeguards reduced the risk of discarding clearly relevant events. The relatively low precision for the Relevant class (57.5%) reflects the intentional design choice to prioritize sensitivity, that is, to minimize missed drowning cases at the cost of a higher false-positive rate. In a surveillance context where expert review of flagged cases is the next step, this trade-off is appropriate: false positives add workload but do not result in missed cases, whereas false negatives represent unretrievable data loss. In practical terms, AquaAI is therefore best understood as a risk-managed triage tool that supports expert review rather than as a fully autonomous classifier.

### Error analysis

The structured expert review adds important nuance to the interpretation of model errors. Although the review was undertaken to better understand why the model had difficulty assigning the correct labels, it also showed that some apparent misclassifications were not straightforward model failures, as the original reference label was not always clearly supported by the narrative on re-review. This suggests that part of the observed error burden reflects ambiguity in the source narratives and challenges in applying the class definitions consistently when descriptions were brief, indirect, or lacking clinical detail, rather than model performance alone.

The review further suggests that the main challenge was subclassification of relevant cases rather than failure to identify clearly relevant events. Confusion between Drowning and Aquatic incident often arose in brief or ambiguous narratives where respiratory impairment or altered consciousness was incompletely documented despite water exposure being present. This indicates that some of the observed confusion reflects limitations of the source data and the operational label boundary rather than model weakness alone.

From a surveillance perspective, this error pattern is consistent with the intended function of the system. AquaAI appeared more likely to retain borderline cases for review than to exclude clearly relevant events, which is consistent with the intended function of the hybrid pipeline. Subclassification outputs should therefore be interpreted as decision support for expert review rather than as definitive case labels.

### Limitations

This study has several limitations. First, AquaAI was evaluated only on records first retrieved by the Danish Drowning Formula. The reported performance therefore reflects the classification stage within a two-step pipeline rather than end-to-end screening of all prehospital narratives. Cases without sufficiently explicit water-related cues may never enter the classification stage [12].

Second, the reference standard was based on manual annotation by trained investigators. Although Drowning annotation followed internationally accepted definitions [3, 4], the structured expert review of misclassified hold-out cases found that in 32 of 121 reviewed cases (26%), the original reference label was not clearly supported by the narrative on re-review. This level of label uncertainty, particularly at the boundary between Drowning and Aquatic incident, means that reported performance metrics may underestimate true model capability, and that subclassification performance should be interpreted with caution. Inter-annotator reliability was not formally assessed, which represents a methodological limitation.

Third, development and evaluation were conducted within a single national prehospital documentation ecosystem using a temporal validation design. While the underlying transformer architecture and hybrid design principles may be transferable, the exact operating threshold, keyword safeguards, retrieval strategy, and class boundaries are likely to be context dependent. Generalizability to other healthcare systems, languages, and documentation styles therefore remains uncertain and should be examined in future studies. Specifically, external application would require re-annotation of training data according to local class definitions, adaptation of the keyword override list to the target language and documentation style, and re-calibration of the Non-relevant threshold against local prevalence. Prospective evaluation in at least one external prehospital setting with independent annotation would be an important next step before broader deployment is considered.

Fourth, the class distribution shifted over the study period, with a decreasing proportion of Drowning cases and an increasing proportion of Non-relevant records in later years (Table 1). This means the Drowning class was somewhat underrepresented in the hold-out dataset relative to the training period, which may affect the generalizability of the reported Drowning sensitivity to future surveillance periods with different case-mix compositions. Whether this shift reflects a true change in drowning incidence, changes in documentation practice, or other factors remains unclear. Regardless of the underlying cause, continued distributional shift may affect model performance over time, particularly for the Drowning class, and periodic revalidation should therefore be considered an integral part of any operational deployment.

Finally, governance restrictions prevent sharing raw narratives, limiting full external reproducibility despite reporting of model architecture, thresholds, and rules. Formal calibration of the predicted probabilities was not evaluated, as the system relies on a fixed threshold rather than direct use of predicted probabilities for clinical decisions.

### Implications for future use

In practical use, AquaAI may support batch-based national surveillance by prioritizing potentially relevant narratives for expert review and generating standardized, audit-ready outputs. External validation in other national or regional prehospital systems, ideally with independent annotation and different documentation languages, would be a necessary step before considering broader implementation. In future routine use, it may support more timely and consistent national drowning surveillance while remaining embedded in expert review workflows.

Deployment of AquaAI in a routine surveillance setting raises important governance considerations. The system is designed to support rather than replace expert judgment, and all records predicted as Relevant are forwarded for human review before inclusion in any surveillance dataset. The keyword override mechanism generates audit flags for all triggered cases, supporting traceability and retrospective review of automated decisions. Nonetheless, any operational use should include defined procedures for handling edge cases and ambiguous classifications, periodic revalidation, and clear institutional accountability for the outputs of the automated triage stage.

## Conclusions

AquaAI, a hybrid transformer-based pipeline operating as the second stage of a two-step surveillance workflow, demonstrated high sensitivity for relevant-case identification from prehospital free-text narratives while substantially reducing the volume of records requiring manual review workload. These findings support the use of AquaAI as a support tool for manual expert validation in a drowning surveillance workflow.

## Supplementary Information

Below is the link to the electronic supplementary material.

Supplementary Material 1: Model specification, preprocessing, and training configuration

Supplementary Material 2: Critical keyword override terms and matching principles

Supplementary Material 3: Year-specific hold-out performance of AquaAI for 2023 and 2024

Supplementary Material 4: Operating threshold evaluation

## Data Availability

The data that support the findings of this study are subject to Danish data protection legislation and are therefore not publicly available. Access may be granted only after approval from the relevant Danish authorities and in accordance with applicable regulations.

## Declarations

### Authors information

The author group is affiliated with TrygFonden’s Drowning Research Unit, Prehospital Center, Region Zealand, and Aalborg University. The author group combines expertise in drowning research, prehospital emergency medicine, epidemiology, and machine learning.

## Abbreviations

AI: Artificial intelligence
AUC: Area Under the Curve
AUC-PR: Area Under the Precision-Recall Curve
AUC-ROC: Area Under the Receiver Operating Characteristic Curve
BERT: Bidirectional Encoder Representations from Transformers
CI: Confidence Interval
EMS: Emergency Medical Services
FP: False Positives
FN: False Negatives
ICD: International Classification of Diseases
NLP: Natural language processing
NPV: Negative Predictive Value
PPV: Positive Predictive Value
TP: True Positives
TN: True Negatives
