# Supplementary material for: AquaAI: development and internal validation of a Danish transformer-based model to identify drowning and aquatic incidents in prehospital medical records
Source: Scand J Trauma Resusc Emerg Med. 2026 Jun 9;34:137. doi: 10.1186/s13049-026-01644-y (PMC13479569; doi:10.1186/s13049-026-01644-y)
Supplement: Supplementary file 1 [file 13049_2026_1644_MOESM1_ESM.docx]

Supplementary Table S1: Model specification, preprocessing, and training configuration

| Item | Specification |
| --- | --- |
| Model backbone | Danish BERT sequence-classification model |
| Exact model identifier | Maltehb/danish-bert-botxo |
| Task | Three-class sequence classification (Non-relevant, Aquatic incident, Drowning) |
| Training framework | Hugging Face Trainer API |
| Tokenizer | Model tokenizer corresponding to Maltehb/danish-bert-botxo |
| Maximum sequence length | 512 tokens |
| Input text construction | Prehospital free-text narrative fields concatenated in a fixed order |
| Input cleaning | Duplicate and empty entries removed |
| Text preprocessing | Minimal, non-destructive preprocessing; no stemming or stop-word removal |
| Long-text handling | Truncation to model input limit before tokenization |
| Temporal split used in final manuscript | Training pool: 2016-2021; validation set: 2022; hold-out dataset: 2023-2024 |
| Training-pool imbalance handling | Random undersampling of the Non-relevant class in the training set only |
| Training-set class sampling used in development | Non-relevant: 5,000; Aquatic incident: 3,204; Drowning: 1,190; total: 9,394 records |
| Validation prevalence | Natural prevalence retained |
| Hold-out prevalence | Natural prevalence retained in the hold-out dataset |
| Loss function | CrossEntropyLoss with class weights |
| Optimizer | AdamW |
| Weight decay | 0.01 |
| Learning rate | 2 × 10^-5 |
| Batch size | 16 |
| Maximum epochs | 10 |
| Early stopping | Yes; patience = 2 epochs |
| Early-stopping target | Validation-set F1-score |
| Evaluation schedule during training | Every epoch |
| Evaluation metric during training | Weighted F1-score |

The table summarizes the technical model specification and development configuration used for AquaAI. The classifier was based on the Danish BERT model Maltehb/danish-bert-botxo and fine-tuned for three-class sequence classification. Training used class-weighted cross-entropy and early stopping based on validation performance.
