# Supplementary material for: AquaAI: development and internal validation of a Danish transformer-based model to identify drowning and aquatic incidents in prehospital medical records
Source: Scand J Trauma Resusc Emerg Med. 2026 Jun 9;34:137. doi: 10.1186/s13049-026-01644-y (PMC13479569; doi:10.1186/s13049-026-01644-y)
Supplement: Supplementary file 2 [file 13049_2026_1644_MOESM2_ESM.docx]

# Supplementary Appendix: Critical keyword override terms and matching principles

*Purpose of the override mechanism*

The critical keyword override was designed as a one-way safety mechanism to reduce the risk of missing drowning cases. It was applied only to narratives initially classified as Non-relevant by the model. If predefined drowning-related terms or phrases were detected, the final prediction was overridden to Drowning. Predictions already assigned to ‘Drowning’ or ‘Aquatic incident’ were not changed.

*Matching strategy*

The override used two complementary matching strategies. Exact matching was performed with case-insensitive regular-expression patterns to identify explicit drowning-related terminology. Fuzzy matching was applied to selected drowning-related words and phrases using RapidFuzz partial string matching with a similarity threshold of 85%, allowing capture of minor misspellings, inflectional variants, and descriptive phrases. All override triggers were logged as audit flags.

Supplementary Table S2: Critical keyword override terms and phrases

| Tier | Matching principle | Terms / phrases (Danish) | Terms / phrases (Translated) |
| --- | --- | --- | --- |
| Exact-match critical terms | Case-insensitive regex matching | *druknet*; *drukning*; *drukne* | *drowned; drowning; drown* |
| Fuzzy/phrasal drowning indicators | RapidFuzz partial string matching (similarity threshold 85%) | *ansigtet under vand;*  *ansigt under vand;*  *drukkenulykke;*  *nærdrukn;*  *død i vand;*  *livløs i vandet;*  *dødfunden i vand;*  *livløs i vandkanten;*  *livløs på vandet;*  *strandvasker;*  *fundet i en å;*  *fundet i havn;*  *fundet i pool;*  *fundet i sø;*  *hevet ind i land;*  *hoved i vandet;*  *hoved under vand;*  *hovedet i vand;*  *redde i land* | *the face under water;*  *face under water;*  *drowning accident;*  *near-drowning;*  *dead in water;*  *lifeless in the water;*  *found dead in water;*  *lifeless by the water;*  *lifeless on the water;*  *washed ashore;*  *found in a stream;*  *found in harbor;*  *found in pool;*  *found in lake;*  *pulled ashore;*  *head in the water;*  *head under water;*  *the head in water;*  *rescue ashore* |

The keyword list was curated by domain experts and implemented as a safety layer to reduce the risk of false-negative drowning classifications. Exact-match terms captured explicit drowning terminology, whereas fuzzy terms captured semantically suggestive wording and descriptive scene phrases. The override was one-directional and did not alter predictions already assigned to Drowning or Aquatic incident. The table presents the Danish terms and phrases used in the override; the English translations are provided to convey the meaning of the Danish expressions and were not used in the implemented solution.
