# Supplementary material for: AquaAI: development and internal validation of a Danish transformer-based model to identify drowning and aquatic incidents in prehospital medical records
Source: Scand J Trauma Resusc Emerg Med. 2026 Jun 9;34:137. doi: 10.1186/s13049-026-01644-y (PMC13479569; doi:10.1186/s13049-026-01644-y)
Supplement: Supplementary file 3 [file 13049_2026_1644_MOESM3_ESM.docx]

# Year-specific hold-out performance of AquaAI for 2023 and 2024

# Supplementary Table S3: Performance of AquaAI on the 2023 hold-out data

**(A)** **Three-way confusion matrix**

| **Total population**  **N = 5,284** |  | **AquaAI prediction** | | |
| --- | --- | --- | --- | --- |
|  |  | **Drowning** | **Aquatic incident** | **Non-relevant** |
| **Reference standard** | **Drowning** | 113 | 13 | 1 |
|  | **Aquatic incident** | 51 | 429 | 49 |
|  | **Non-relevant** | 109 | 472 | 4,047 |

**(B)** **Relevant versus Non-relevant confusion matrix**

| Total population  N = 5,284 |  | AquaAI prediction | |
| --- | --- | --- | --- |
|  |  | **Relevant** | **Non-relevant** |
| Reference standard | **Relevant** | TP = 606 | FN = 50 |
|  | **Non-relevant** | FP = 581 | TN = 4,047 |

Rows show the reference standard and columns the AquaAI prediction. Panel (A) shows the three-class confusion matrix for Drowning, Aquatic incident, and Non-relevant. Sensitivity was 89.0% for Drowning, 81.1% for Aquatic incident, and 87.4% for Non-relevant. Panel (B) collapses Drowning and Aquatic incident into Relevant versus Non-relevant. Sensitivity for Relevant was 92.4%, and specificity for Non-relevant was 87.4%.

# Supplementary Table S4: Performance of AquaAI on the 2024 hold-out data

**(A)** **Three-way confusion matrix**

| **Total population**  **N = 5,490** |  | **AquaAI prediction** | | |
| --- | --- | --- | --- | --- |
|  |  | **Drowning** | **Aquatic incident** | **Non-relevant** |
| **Reference standard** | **Drowning** | 133 | 33 | 0 |
|  | **Aquatic incident** | 25 | 600 | 85 |
|  | **Non-relevant** | 73 | 378 | 4,163 |

**(B)** **Relevant versus Non-relevant confusion matrix**

| Total population  N = 5,490 |  | AquaAI prediction | |
| --- | --- | --- | --- |
|  |  | **Relevant** | **Non-relevant** |
| Reference standard | **Relevant** | TP = 791 | FN = 85 |
|  | **Non-relevant** | FP = 451 | TN = 4,163 |

Rows show the reference standard and columns the AquaAI prediction. Panel (A) shows the three-class confusion matrix for Drowning, Aquatic incident, and Non-relevant. Sensitivity was 80.1% for Drowning, 84.5% for Aquatic incident, and 90.2% for Non-relevant. Panel (B) collapses Drowning and Aquatic incident into Relevant versus Non-relevant. Sensitivity for Relevant was 90.3%, and specificity for Non-relevant was 90.2%.
