# Supplementary material for: AquaAI: development and internal validation of a Danish transformer-based model to identify drowning and aquatic incidents in prehospital medical records
Source: Scand J Trauma Resusc Emerg Med. 2026 Jun 9;34:137. doi: 10.1186/s13049-026-01644-y (PMC13479569; doi:10.1186/s13049-026-01644-y)
Supplement: Supplementary file 4 [file 13049_2026_1644_MOESM4_ESM.docx]

**Operating threshold evaluation**

Supplementary Table S5: Threshold comparison on the validation dataset

| Operating configuration | Drowning → Non-relevant (n) | Aquatic incident → Non-relevant (n) | Non-relevant → Relevant (n) | Sensitivity | Specificity | Predicted negative rate | Youden index |
| --- | --- | --- | --- | --- | --- | --- | --- |
| Argmax | 13 | 66 | 348 | 90.3% | 92.4% | 80.0% | 0.827 |
| Threshold 0.50 | 13 | 65 | 353 | 90.4% | 92.3% | 79.9% | 0.827 |
| Threshold 0.90 | 6 | 33 | 542 | 95.2% | 88.2% | 75.7% | 0.834 |
| Threshold 0.95 | 5 | 26 | 667 | 96.2% | 85.5% | 73.2% | 0.817 |

Rows summarize threshold-dependent performance on the validation set (N = 5,409). Relevant sensitivity was calculated after collapsing Drowning and Aquatic incident into Relevant versus Non-relevant. Specificity refers to the proportion of Non-relevant records correctly classified as Non-relevant. Predicted negative rate reflects the proportion of all records classified as Non-relevant by AquaAI and thus removed from manual review ((TN+FN)/N). Youden index = Sensitivity + Specificity − 1. Thresholds Argmax, 0.50, 0.90, and 0.95 were evaluated on the validation set, and 0.90 was selected as the final operating threshold based on the highest Youden index (0.834).

Supplementary Figure S1. ROC and precision-recall curves for threshold evaluation on the validation dataset. Receiver operating characteristic (ROC) and precision-recall (PR) curves for binary Relevant versus Non-relevant classification on the validation dataset (2022, N = 5,409). Panel (A) shows the ROC curve; the dashed diagonal line represents a random classifier. Panel (B) shows the precision-recall curve; the dashed horizontal line represents the no-skill baseline equal to the Relevant class prevalence (15.0%). Operating points for four threshold configurations are shown in both panels and described in the figure legend. Threshold 0.90 was selected as the final operating threshold based on the highest Youden index on the validation set (J = 0.834). AUC-ROC and AUC-PR are reported with 95% bootstrap confidence intervals (2,000 iterations). Threshold-specific performance metrics are summarized in Supplementary Table S5.
